# Shell-to-Beam Numerical Homogenization of 3D Thin-Walled Perforated Beams

**DOI:** 10.3390/ma15051827

**Published:** 2022-02-28

**Authors:** Natalia Staszak, Tomasz Gajewski, Tomasz Garbowski

**Affiliations:** 1 Doctoral School, Department of Biosystems Engineering, Poznan University of Life Sciences, Wojska Polskiego 28, 60-637 Poznan, Poland; natalia.staszak@up.poznan.pl; 2 Institute of Structural Analysis, Poznan University of Technology, Piotrowo 5, 60-965 Poznan, Poland; tomasz.gajewski@put.poznan.pl; 3 Department of Biosystems Engineering, Poznan University of Life Sciences, Wojska Polskiego 50, 60-627 Poznan, Poland

**Keywords:** numerical homogenization, thin-walled steel structure, perforation, beam element, shell element, open cross-section, finite element analysis

## Abstract

Determining the geometric characteristics of even complex cross-sections of steel beams is not a major challenge nowadays. The problem arises when openings of various shapes and sizes appear at more or less regular intervals along the length of the beam. Such alternations cause the beam to have different stiffnesses along its length. It has different bending and shear stiffnesses at the opening point and in the full section. In this paper, we present a very convenient and easy-to-implement method of determining the equivalent stiffness of a beam with any cross-section (open or closed) and with any system of holes along its length. The presented method uses the principles of the finite element method (FEM), but does not require any formal analysis, i.e., solving the system of equations. All that is needed is a global stiffness matrix of the representative volumetric element (RVE) of the 3D representation of a beam modeled with shell finite elements. The proposed shell-to-beam homogenization procedure is based on the strain energy equivalence, and allows for precise and quick determination of all equivalent stiffnesses of a beam (flexural and shear). The results of the numerical homogenization procedure were compared with the existing analytical solution and experimental results of various sections. It has been shown that the results obtained are comparable with the reference results.

## 1. Introduction

Load-bearing members of structures often require regular perforations/holes or uniquely placed openings. This requirement may arise from various causes. One reason is for carrying out installations, such as with electrical wiring or fire sprinkler systems [1]. Going towards more and more smart buildings, the number of wiring systems will increase in the coming years. Another reason for these features is to fix the mounting to the load bearing element, which was analyzed, for example, in [2]. The mounting holes may be localized in specific locations (pre-planned) or in periodic manner (enabling adjustment of the mounting location in situ). In the former type, the cross-section is weakened in a single location, while in the latter type the cross-section has variable and reduced stiffness along its length. Periodic perforations/holes may be also beneficial if the structure mass must be reduced. If at the same time the load bearing capacity needs to be kept at a certain level, optimization techniques are often required [3,4,5].

Computing the properties of the structural member for a constant cross-section is a typical structural/mechanical engineering problem that is easy to solve. However, if a structure member with regular holes or uniquely placed openings is used, the common approach is not sufficient. For the constant cross-section, even with complicated, multi-material assembly, the problem may be solved analytically if an elastic range is considered. The problem is complicated if non-elastic behavior is considered, because in such cases the cross-section properties are not enough to determine the behavior of the structure. In such cases, the General Nonlinear Constitutive Law (GNCL) can be used, as for instance in these works [6,7,8,9]. On the other hand, if the cross-section of the beam member is not constant along its length, additional averaging techniques, section division or individual analytical solutions (applicable to unique type of cross-sections) are required. An analytical approach to solve such problems is highly impractical, since for most engineering applications the solution must be quickly obtained and replicable also to different structures. Additionally, it is known that analytical approaches are frequently not possible, because the problem formulation is too complicated, or the time resources spent to find the solution exceed the benefits. Therefore, the numerical methods, such as the finite element method (FEM) popular among structural engineers, seem to be the approaches that best address the problem.

However, for detailed geometries, the classical finite element method is not cost effective. As an alternative, the extended approach with simple homogenization technique can offer the benefits of the numerical approach in an engineering practice. Classical FEM is not effective, because the member with regular holes or unique openings would require modelling it via solid or at least shell elements [1,2,10]. Such modelling is computationally costly, mainly due to the need for using detailed meshes, especially when the element contains regular holes along its length. Additionally, the solid or shell elements must be available in an in-home or commercial FEM code, which is not straightforward to implement and not common in typical engineering software. Extending the FEM with homogenization technique for structural members certainly could simplify the problem.

Homogenization techniques are used to solve complex mechanical problems in structures for many years. Various approaches have been used, for instance in [11], for lattice beam-like structures, a direct approach based on the matrix eigenvectors and principal vectors of the state transfer matrix was utilized. In [12], the periodic beam-like structure homogenization was presented. The homogenization was obtained by the authors from the solution of basic cell problems posed on the three-dimensional period of the structure, and solved using a three-dimensional finite element method. The homogenization of helical beam-like structures in a two-dimensional model were analyzed in [13] under axial loads (extension or torsion). This approach was extended in [14], in which the helical beam-like structures under bending loads were considered. Homogenization in beams enables consideration of the Timoshenko theory, for instance in [15], in which the homogenization of a 3D model of a non-centrosymmetric tetrachiral unit cell was investigated as an example of architected heterogeneous material.

Among other scientists who have recently made their contribution to the development of homogenization techniques, one should mention [8,16,17,18]. In [16], the plate homogenization of a shell structure to a single shell element was proposed, as an extension of Biancolini’s approach [19]. In [17], the civil engineering structures of prefabricated composite floor-slabs, the so-called “Filigree” slabs, were considered, in which homogenization was also used to determine floor-slab material properties, but also their deflections. Other types of structures were also considered, for instance, composite steel and concrete “I” beams [6] or steel frames [7]. In [18], the method of determining the stiffness properties of perforated corrugated cardboard was shown, using the homogenization technique proposed earlier by Garbowski & Gajewski in [16]. In all of these cases, the homogenization technique was a useful tool to speed up numerical estimation, determine the structure properties or simplify the FEM model.

When applying homogenization techniques, one eliminates the analytical and classical FEM drawbacks, i.e., nonexisting solutions, overcomplex problems or costly solutions. Homogenization techniques enable the modelling of very detailed geometries, for instance with regular holes along the member [20,21]. It should be underlined that often the structures are successfully considered as full-shell finite element (FE) models with elaborate geometric complexity; for instance, see [2,22,23]. However, applying homogenization techniques enable speeding up of the computations, for instance in optimization processes [21,24]. Optimization using homogenization may be used to determine the parameters of the structure, both material [25,26] or geometric [27,28], or its topology [24,29]. The homogenization technique presented in the forthcoming section may be used in all such optimization problems. In this paper, computing the structural and material properties of steel members with regular holes was shown as the example of the application of the homogenization techniques proposed.

The paper’s main objective is to present a novel shell-to-beam numerical homogenization method based on deformation energy equivalence. The derivation and application of a numerical homogenization technique are demonstrated in thin-walled beams with periodic openings. The Materials and Methods chapter consists of two sections. In the first section, the numerical models used are described, while in the second the mathematical details of the homogenization method are explained. The Results chapter contains mainly a comparison with the analytical approach and nominal numerical examples of the homogenization technique. In the first section of this chapter, Z and C profiles without any holes were considered, while in the second section, more elaborate cases were used, i.e., one with the holes in the web and rounding in the corners. In the Discussion chapter, the results obtained are comment upon in detail, and the references to the experimental results from the literature were also investigated. Good agreement of the results with the reference data was obtained in all examples presented.

## 2. Materials and Methods

### 2.1. Beams—Numerical Models

The thin-walled cold-formed profiles of Z, C and square tube are the numerical examples considered in this paper. Thin-walled cold-formed profiles are cost-effective modern building members used in light structures. Apart from economics, their main advantage is quick and effective forming of wall and roof purlins in such facilities as industrial buildings, warehouses, commercial buildings, livestock structures and production halls. However, these kinds of members are vulnerable to initial imperfections and local instability [30,31]. As already noted in previous section, the openings are often necessary for use in roof elements, especially to carry out installations. However, these kinds of members are beneficial in a structure, because they may be designed for an optimal distribution of weight and geometry of cross-section to attain particular strength and load-carrying capacity. Their lightness greatly facilitates assembly in situ.

**Figure 1:**
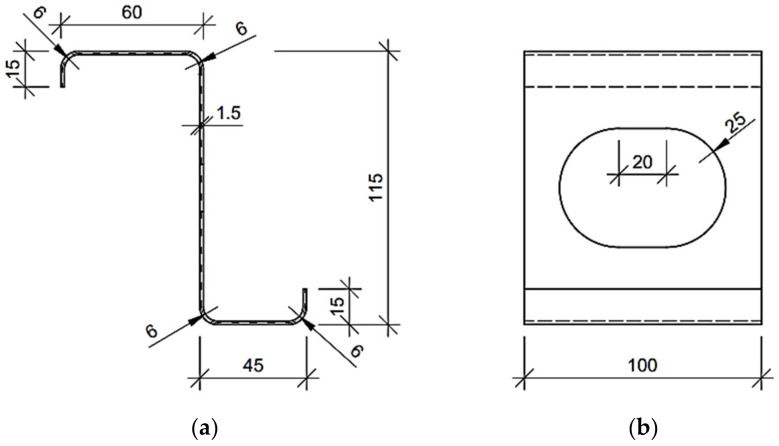
Considered Z profile with regular stadium hole in the web (units in mm): (**a**) cross-section and (**b**) side view on representative volume element.

**Figure 2:**
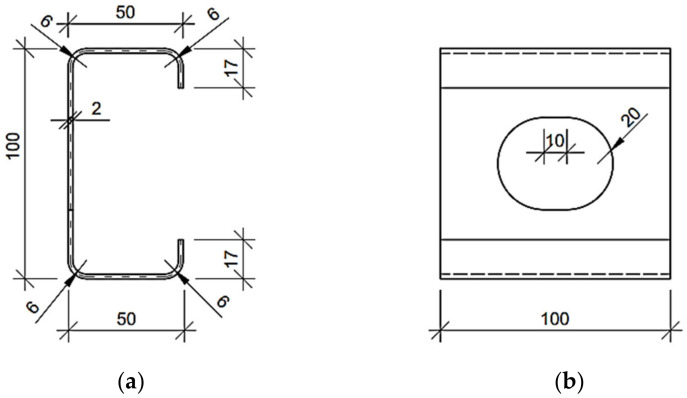
Considered C profile with regular stadium hole in the web (units in mm): (**a**) cross-section and (**b**) side view on representative volume element.

**Figure 3:**
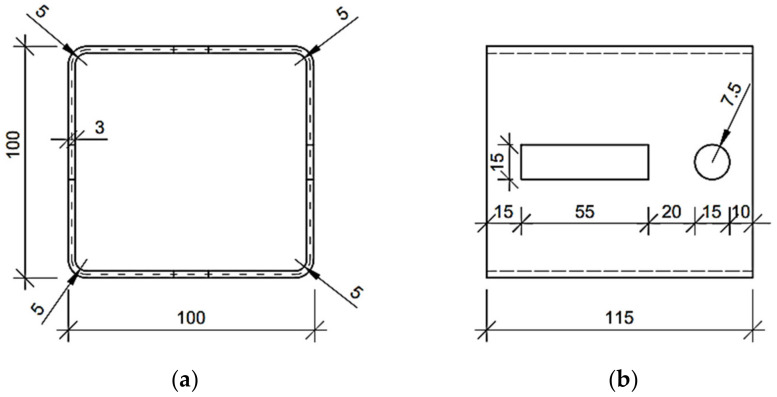
Considered square tube with regular mounting holes in the web (units in mm): (**a**) cross-section and (**b**) side view on representative volume element.

The numerical examples of the Z, C and square tube profiles considered here may be divided into three categories: (i) no corner-rounding and no holes, (ii) corner-rounding and no holes, and (iii) both corner-rounding and periodic holes. The examples from the (iii) category are presented in Figure 1, Figure 2 and Figure 3, with all cross-sections and out-of-plane dimensions of representative volume element (RVE) used in the later computations. In the (ii) category, the holes in the web are not considered. In the (i) category, the four rounding of the corners are not defined (i.e., the corners are exactly 90 degrees). For each example of the Z or C profiles, the stiffeners are assumed and included in computations.

In order to build the full stiffness matrix, $K_{e}$, of the RVE of selected examples, the Abaqus FEA code was used [32].

**Figure 4:**
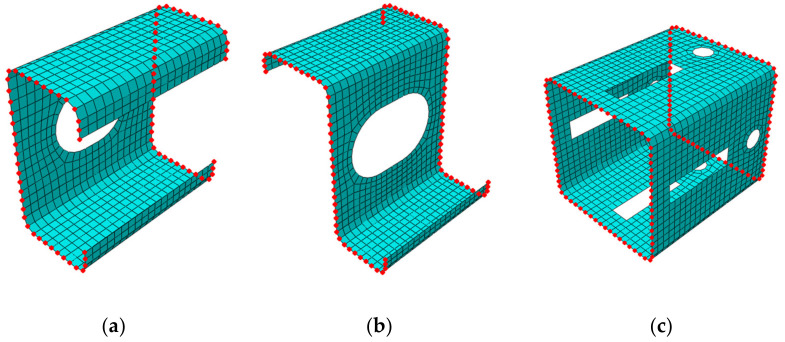
Computational examples of representative volume elements with nodes selected, to which the entire stiffness is statically condensed: (**a**) C profile; (**b**) Z profile and (**c**) square tube.

In Figure 4, the selected meshes of RVEs are presented for the numerical examples considered. The red nodes denote the external nodes for static condensation; for details, see Section 2.2. The element type used for forming a full stiffness matrix K for RVE was a 4-node general-purpose shell element (named S4 according to Abaqus FEA Documentation [32]). In all numerical examples, the mesh size varied from 2.5 mm to 15 mm. Therefore, for the Z profile without holes, no corner-rounding and elongation depth equal to 100 mm, the number of nodes (elements) was 15,520 (3880) for 2.5 mm seed and 476 (119) for 15 mm seed. For the C profile with a length of 100 mm belonging to the (i) category example, the number of nodes (elements) was 14,240 (3560) for 2.5 mm seeds and 420 (105) for 15 mm seeds. Profiles from the (ii) and (iii) categories were computed only for the 5 mm seed size. Such discretization gives 824 elements (3296 nodes) for the Z profile with holes (Figure 4a), 781 elements (3124 nodes) for the C profile with holes (Figure 4b) and 1541 elements (6164 nodes) for the square tube with holes (Figure 4c). The isotropic linear elasticity was used to describe the mechanical behavior of steel. The following material parameters were assumed: the Young’s modulus equal to E=210 GPa and the Poisson’s ratio equal to $\nu =0.3\;\left[–\right]$.

In this work, for computing the reference results used in the verification section (see Section 3.1), the typical engineering approach for simple cross-sections from the (i) category examples were used to compute the profile’s stiffnesses analytically. This means that the reference models used for the verification of the method did not contain holes and had a simplified geometry (the rounding of the corners were neglected) so that it was possible to easily determine their geometric characteristics (i.e., moments of inertia). The basic analytical formulas, which can be found in the strength of materials textbooks, were used to calculate the stiffness of the cross-sections selected, for instance $EI_{x}=E\int \int_{A}y^{2}dA$ and $EI_{y}=E\int \int_{A}x^{2}dA$. The geometric properties, such as widths, heights and eccentricity from the neutral axis of the cross-sections used in the study are presented in the Figure 1, Figure 2 and Figure 3.

### 2.2. Shell-to-Beam Numerical Homogenization

Homogenization is often used for a significant simplification of the computational model, which in turn saves computational time for the given problem. This is especially important in the case of 3D models that contain inclusions, cavities, pores or dispersed reinforcement. Modeling of such structures requires the use of advanced meshing techniques as well as careful and laborious partitioning techniques in order to properly assign the appropriate mechanical features to different parts of the model.

**Figure 5:**
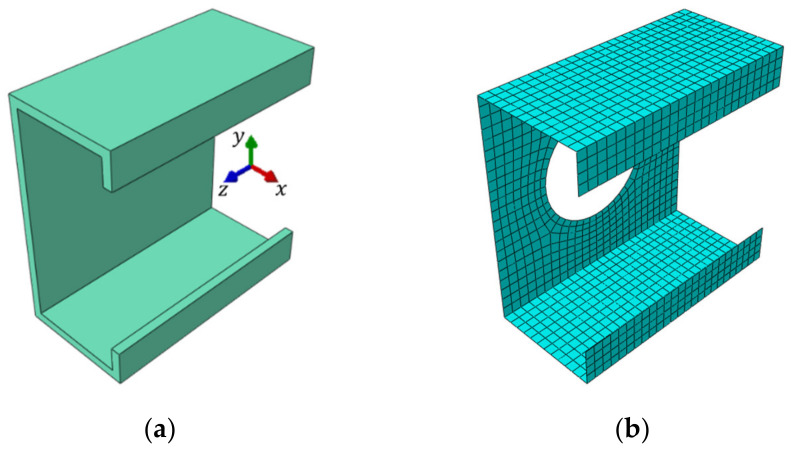
(**a**) Cross-section of structural beam model and (**b**) 3D shell model.

In the case of beam structures, which can be modeled as structural elements, it is usually assumed that the cross-section is made of one type of material, and the element itself does not contain any local weaknesses along its length. Therefore, for the correct geometric description of structural elements, the moments of inertia in relation to two mutually perpendicular axes (i.e., *x* and *y* axis) and the cross-sectional area as well as material data are sufficient (see Figure 5a).

If the cross-section of the beam is complex, for example consisting of multiple materials or with any longitudinal openings, the numerical model usually consists of 3D finite elements—both shell and/or solid (see Figure 5b). This type of modeling gives more accurate results, but makes the analysis much longer. A tool that can be used to shorten the analysis and at the same time maintain the accuracy of the results is the aforementioned homogenization [16,17,18].

In the case of thin-walled beam structures, the material from which they are built is usually homogeneous, and the cross-section itself is not composed of many different materials. However, the appearance of holes along the beam may quickly turn a simple analysis into a complex task. Thus, in order to avoid complicated modeling, numerical homogenization can be used [16,17,18,19,33]. The homogenization method proposed in this paper is an adaptation of an existing technique based on deformation energy equivalence [19]. This method was previously used for layered shell sections [16,18] as well as for concrete slabs reinforced with spatial trusses [17].

**Figure 6:**
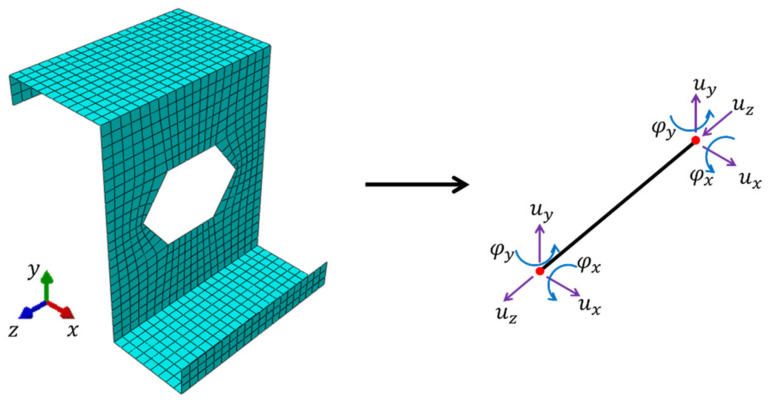
Shell-to-beam transformation.

The main step in this approach is to construct a deformation–displacement relationship in order to apply elastic strain energy equivalence between the full 3D model (homogenized) and the model reduced to a structural element (see Figure 6).

In order to compare both models (i.e., the full 3D and the structural model), the first step is to condense the stiffness of the full model to the nodes, in which the kinematic boundary conditions are applied. For this purpose, the global stiffness matrix is subject to static condensation (i.e., the overall RVE stiffness is condensed to selected nodes, see Figure 4).

The nodes to which the stiffness of the entire RVE is condensed are located at the front and rear of the cross-section (see Figure 4). The condensed stiffness matrix can be computed from the standard formula:

(1) $$K_{e}=K_{ee}-K_{ei}\;K_{ii}{}^{-1}\;K_{ie},$$

where the subarrays are related to the external (subscript e) and internal (subscript i) nodes:

(2) $$K\;u=F\;\to \;\left[\begin{array}{cc} K_{ee} & K_{ei}\\ K_{ie} & K_{ii} \end{array}\right]\left[\begin{array}{c} u_{e}\\ u_{i} \end{array}\right]=\left[\begin{array}{c} F_{e}\\ 0 \end{array}\right].$$

The total elastic strain energy can be expressed as the work of external forces on the corresponding nodal displacements:

(3) $$E=\frac{1}{2}\;u_{e}^{T}\;F_{e}.$$

Substituting nodal forces vector with classical FE formulation (2) the strain energy can be expressed by the following formula:

(4) $$E=\frac{1}{2}u_{e}^{T}K_{e}u_{e}.$$

By introducing the strain-displacement transformation matrix, $H_{e}$, which links strains with translations and rotations in all external nodes:

(5) $$u_{e}=H_{e}\varepsilon_{e},$$

the strain energy reads:

(6) $$E=\frac{1}{2}\varepsilon_{e}^{T}\;H_{e}^{T}K_{e}H_{e}\varepsilon_{e},$$

which can be finally simplified to:

(7) $$E=\frac{1}{2}\varepsilon_{e}^{T}\;H_{k}\;\varepsilon_{e}\left\{length\right\},$$

where $H_{k}$ is a matrix consisting of all sought compression, bending and shearing stiffnesses:

(8) $$H_{k}=\frac{H_{e}^{T}\;K_{e}\;H_{e}}{\left\{length\right\}}=\left[\begin{array}{ccccc} A_{33} & B_{31} & B_{32} & 0 & 0\\ B_{13} & D_{11} & 0 & 0 & 0\\ B_{23} & 0 & D_{22} & 0 & 0\\ 0 & 0 & 0 & R_{44} & 0\\ 0 & 0 & 0 & 0 & R_{55} \end{array}\right],$$

where $A_{33}$ is the tensile/compression stiffness along *z* axis, $D_{11}$ and $D_{22}$ are the bending stiffnesses with respect to the axes 1 and 2 (i.e., *x* and *y* axis), $R_{44}$ and $R_{55}$ are the shear stiffnesses of RVE, while $B_{13}=B_{31}$ and $B_{23}=B_{32}$ are the compressive-bending terms. If $B_{13}$ and/or $B_{23}$ are present in the $H_{k}$ matrix, it means that the homogenized RVE was not aligned with the natural axes. In such case, in order to determine the bending stiffness $D_{ii}^{*}$ in the neutral axes, one should use the simple relationship to replace $D_{11}$:

(9) $$D_{11}^{*}=D_{11}-\frac{B_{13}^{2}}{A_{33}},$$

and $D_{22}$:

(10) $$D_{22}^{*}=D_{22}-\frac{B_{23}^{2}}{A_{33}}.$$

The heart of the method is the transformation matrix $H_{i}$ determined for each node ($x_{i}=x$, $y_{i}=y$, $z_{i}=z$), which defines the relationship between nodal displacements and the nodal state of deformation responsible for unit elongation, bending or shearing:

(11) $$u_{i}=H_{i}\;\varepsilon_{i},$$

or:

(12) $${\left[\begin{array}{c} u_{x}\\ u_{y}\\ u_{z}\\ \theta_{x}\\ \theta_{y} \end{array}\right]}_{i}={\left[\begin{array}{ccccc} 0 & 0 & -z^{2}/2 & z/2 & 0\\ 0 & -z^{2}/2 & 0 & 0 & z/2\\ z & yz & xz & x/2 & y/2\\ 0 & 0 & -z & 0 & 0\\ 0 & -z & 0 & 0 & 0 \end{array}\right]}_{i}{\left[\begin{array}{c} \varepsilon_{z}\\ \kappa_{x}\\ \kappa_{y}\\ \gamma_{xz}\\ \gamma_{yz} \end{array}\right]}_{i}.$$

Thus, the complete shell-to-beam homogenization procedure consists of the following steps:

1. Build a full stiffness matrix K;
2. Static condensation of the stiffness matrix to the outer nodes (e), in which the boundary conditions are applied—computing $K_{e}$ from Equation (1);
3. Determination of a transformation matrix $H_{i}$ for each outer node from Equation (12);
4. Assemble the matrix $H_{e}$;
5. Determination of the matrix $H_{k}$ containing the searched RVE stiffnesses reduced to the structural element.

It is worth noting that in the above procedure, no formal FEM analysis is carried out, i.e., solving the system of equations, only simple matrix operations are required that lead to designated stiffnesses in a single step. In the traditional homogenization approach, all deformation states are applied in a sequence of steps in order to obtain all sought effective stiffnesses.

## 3. Results

### 3.1. Verification of Numerical Homogenization with an Analytical Approach

This section presents the results obtained from numerical analyses (numerical homogenization) and an analytical approach for profiles from the (i) category example. The calculations were made for the C and Z profile without holes and no corner-rounding, depending on the elongation depth and mesh seed size. Table 1 shows the stiffness for the Z profile with constant mesh size equal to 5 mm, due to the variable elongation depth of this model. The elongation depth ranged from 5 mm to 400 mm. The last row in the table presents the analytically determined stiffnesses.

**Figure 7:**
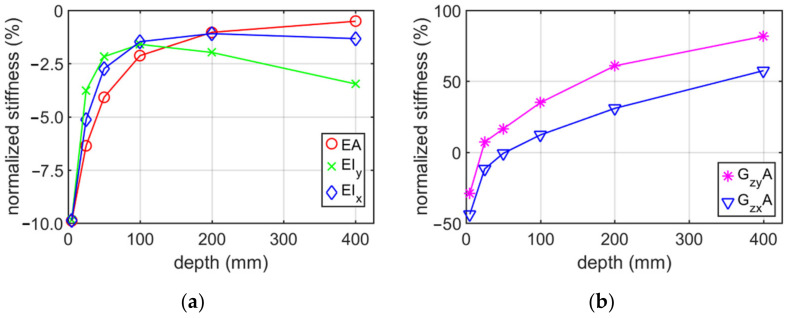
Plots of normalized stiffness of Z profile (without holes and no corner-rounding) with 5 mm mesh, depending on the elongation depth (beam axis): (**a**) bending and tensile/compression stiffnesses; (**b**) shear stiffness.

Figure 7 shows the normalized stiffness of the Z profile without holes and no corner-rounding, depending on the elongation depth (beam axis). The normalized values were calculated by dividing the numerical values of stiffnesses via their analytical counterparts.

**Figure 8:**
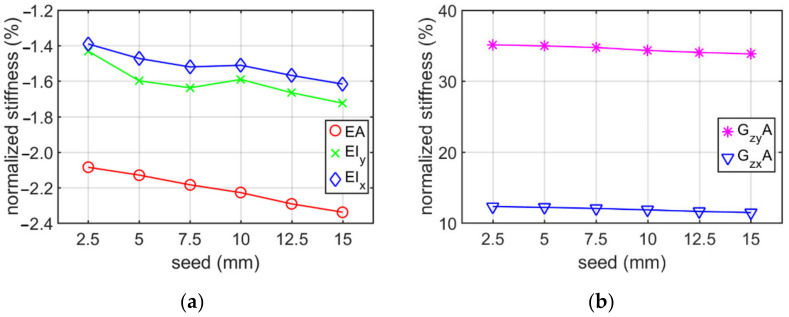
Plots of normalized stiffness of Z profile (without holes and no corner-rounding) with constant elongation depth of 100 mm, depending on mesh seed: (**a**) bending and tensile/compression stiffnesses; (**b**) shear stiffness.

The change in stiffness due to mesh seed for the Z profile belonging to the (i) category example with constant elongation depth of 100 mm is presented in Table 2. The mesh seed was assumed to vary from 2.5 mm to 15 mm. Figure 8 shows the normalized values of stiffness calculated on the basis of Table 2.

Table 3 and Table 4 show the comparison of the stiffness of the C profile without holes and no corner-rounding obtained from the numerical homogenization and analytical approach. The values of stiffness for the profile with 5 mm mesh seed and variable elongation depth are presented in Table 3.

**Figure 9:**
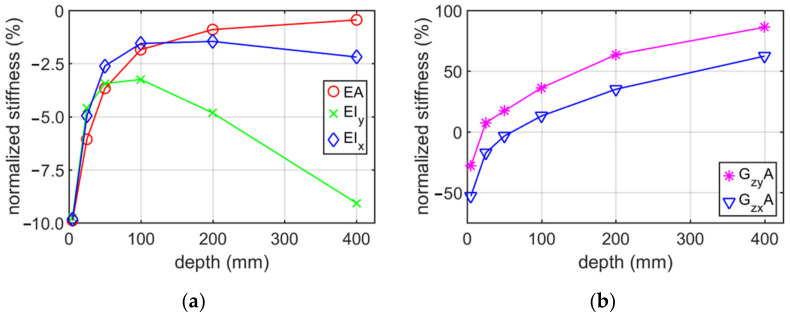
Plots of normalized stiffness of C profile (without holes and no corner-rounding) with 5 mm mesh, depending on the elongation depth: (**a**) bending and tensile/compression stiffnesses; (**b**) shear stiffness.

Figure 9 presents the normalized stiffness of the C profile for different elongation depths from 5 mm to 400 mm and mesh seed equal to 5 mm.

Table 4 lists the stiffness due to the mesh size (2.5–15 mm) for the C profile with no corner-rounding and without holes. The elongation depth was constant and equal to 100 mm.

**Figure 10:**
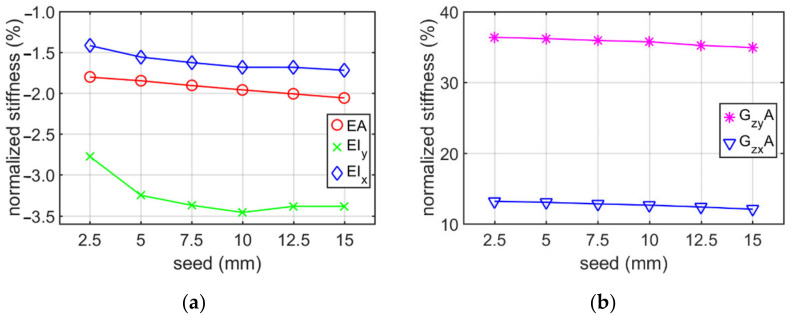
Plots of normalized stiffness of C profile (without holes and no corner-rounding) with constant elongation depth of 100 mm, depending on mesh seed: (**a**) bending and tensile/compression stiffnesses; (**b**) shear stiffness.

In addition, the normalized stiffness for this case are presented in Figure 10.

### 3.2. Numerical Homogenization

In the next step, the numerical homogenization was used to compute profiles from examples of the (ii) and (iii) categories i.e., Z profile, C profile and square tube with rounding, were considered. The comparison of stiffnesses obtained from those profiles with hole and without hole is presented in Table 5.

**Figure 11:**
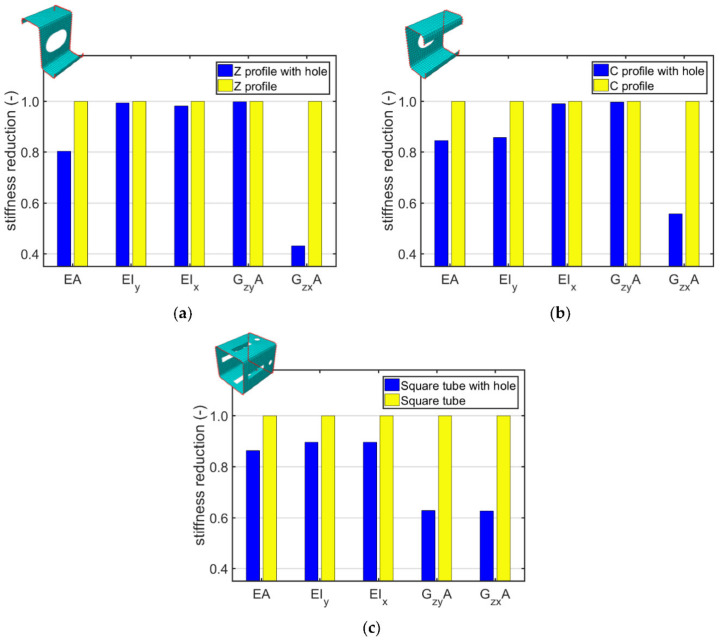
Stiffnesses reductions computed by homogenization technique for: (**a**) Z profile, (**b**) C profile and (**c**) square tube without and with holes.

Figure 11 shows the stiffness reductions calculated by the numerical homogenization for profiles with rounding, in which periodic holes were assumed in comparison to the cases without the holes. Z profile (Figure 11a), C profile (Figure 11b) and square tube (Figure 11c) with hole and without hole were investigated.

## 4. Discussion

The conducted analyses allowed to obtain information what is the influence of the RVE depth and mesh size on the stiffness of a thin-walled beam for the cross-sections considered. Additionally, the influence of holes on the stiffness was studied. Table 1, Table 2, Table 3 and Table 4 show the stiffness of C and Z profile without hole and no corner rounding obtained from numerical homogenization and analytical formulas. The change in the stiffness depending on different elongation depth and constant mesh size are presented in Table 1 and Table 3. The estimation error of the tensile/compression stiffnesses are the smaller, the greater is the elongation depth. The estimation error reaches about −0.5% for an elongation of 400 mm. The bending stiffnesses have the lowest error values for RVE depth approximately 100 mm. In the whole range of elongations considered it varied from −10% up to −2%. Therefore, the elongation depth equal to 100 mm was assumed in Section 3.2. Moreover, it can be observed that the greatest difference in stiffnesses occurred for the shear stiffness with relation to elongation change, i.e., from −53% up to 86%. The optimal position (lowest value of error) was obtained for the RVE depth between 25 and 50 mm. The results presented in Table 2 and Table 4 show the stiffness profile (i) category example due to the various mesh size. Based on these data, it can be seen that the change of normalized stiffness depending on the seed achieved a maximum at about 0.5 % (see Figure 8 and Figure 10). The normalized value of stiffness reached a value of several percent for tensile or bending stiffness, dozen percent for shear stiffness in the web plane and several dozen (about 37%) for shear stiffness in the plane of the shelf.

Based on results presented in Section 3.1, it can be observed that the choice of the elongation depth had a much greater impact on the error of stiffness estimation than the adopted mesh size. Therefore, it is advised to perform preliminary mesh dependence study in order to avoid elevated error, while using the homogenization technique presented.

The analyzes presented in the Section 3.2 concern profiles with rounded corners and both with and without a hole. In all cases analyzed, considering the holes essentially decreases the tensile/compression stiffnesses, from 14% for square tube up to 20% for Z profile (see Figure 11). The bending stiffness of Z profile with hole did not differ from that of full cross-section because the hole is close to the center of gravity (Figure 11a). In C profile, the decrease of bending stiffness is severe since the web has meaningful eccentricity from the neutral axis. In square tube, the decrease of bending stiffness in both directions is exactly the same, this outcome was expected since the profile has a double symmetry of the cross-section. Moreover, the drop in shear stiffness for this profile was the same in both planes and amounted to 37%. On the other hand, shear stiffness in the plane of the shelf for C and Z profiles with hole did not change because the hole is located in the web. Therefore, the shear stiffness in this plane decreases by 57% for Z profile and 44% for C profile. In addition, the stiffness was influenced by the size of the hole.

The homogenization method presented was also verified with the experimental results from the literature, namely, with the study of Nawar et al. [34]. In the study, the static resistance of castellated steel I beams with hexagonal web openings were considered. Load-deflection relationships for two types of specimens (labelled as CB-01 and CB-04) from bending by applying two concentrated forces were reported. The initial part of the experimental plots, i.e., an elastic range, were used by us to compute the bending stiffnesses of those samples, which were equal to $EI_{x}=1.6\times 10^{12}\;{\mathrm{MPa}\;\mathrm{mm}}^{4}$ for CB-01 and $EI_{x}=2.7\times 10^{12}\;{\mathrm{MPa}\;\mathrm{mm}}^{4}$ for CB-04. The bending stiffnesses from homogenization techniques presented in this study were equal to $EI_{x}=1.05\times 10^{12}\;{\mathrm{MPa}\;\mathrm{mm}}^{4}$ for CB-01 and $EI_{x}=3.2\times 10^{12}\;{\mathrm{MPa}\;\mathrm{mm}}^{4}$ for CB-04. It is worth noting, that due to poor resolution of the plot, in which our region of interest is a few percent of the whole horizontal axis, the digitalized data may be burdened with error. Moreover, the test replicates the portal frame structure, thus, the end of the beams tested are not exactly the fixed ends, therefore the deflection registered may be disturbed. Taking into account the above considerations, it can be concluded that the results are in good agreement.

The authors are aware of a few limitations of the work. Namely, nonlinear material properties cannot be taken into account, and therefore only the elastic response of the beam can be determined (plasticity or failure in the material response cannot be considered). Moreover, a discretization of the FE model used is crucial to receive trustworthy results of the numerical homogenization technique proposed. In addition, the periodicity of the beam enforces the RVE depth in beam axial direction, which may not allow finding the optimal FE mesh for reliable results.

The homogenization method derived can be also used in the different engineering disciplines for example to find optimal cross-sections for particular load-bearing elements. This technique may be used e.g., in computer aided design of optimal purlins or trusses in structural engineering, road crush barriers in civil engineering or light mounting rails in environmental engineering, etc. Using homogenization technique proposed can greatly reduce the computational time which is always crucial in such optimization problems. In academia, the method has the potential to be still developed by adding more peculiar conditions, such as periodic boundary conditions, or including nonlinear effects (i.e., plasticity, damage) and many others. According to the best knowledge of the authors, the homogenization method used (both in this article but also in the previous ones [16,17,18]) is the first to use a condensed computational approach that does not require systematic reconstruction of all deformation states of the analyzed element, and thus does not require formal FEM analyzes such as tension, compression, shear and bending of the RVE.

## 5. Conclusions

In the paper, the shell-to-beam homogenization technique was developed and presented. The homogenization technique proposed has been adapted from the already existing technique based on elastic deformation energy equivalence. The proposed extension of the homogenization method allows to compute effective stiffnesses of the 3D thin-walled beam with periodic openings along its length, which drastically simplifies computations. The main idea utilizes constructing a deformation-displacement relationship which represents an elastic strain energy equivalence of the full 3D model (homogenized) by the simplified model (reduced to a structural element).

The effectiveness of the technique proposed was confirmed by three numerical examples of thin-wall cold-formed Z and C profiles, as well as square tube by the comparison of the numerical outcomes of the homogenization with their analytical counterparts. One of the main advantage of the homogenization technique proposed is evidenced in perforated beam sections, which are costly to be modelled via traditional finite element models or too complex to get a solution analytically. In the paper, it was proved by the numerical examples that the technique is adequate to be used in perforated beams with local or regular holes/openings in the thin-wall profiles, but is not limited to such. The technique may be extended to be used in other types of structural members, such as hot rolled sections, wooden elements or encased sections. Future work would be devoted to including imperfections in the homogenization technique with fuzzy probability.

## Figures and Tables

**Table 1:** Stiffness of Z profile (without holes and no corner-rounding) with 5 mm mesh depending on the elongation depth (beam axis).

Depth $\left(mm\right)$	EA $\left(10^{7}\;MPa\;mm^{2}\right)$	$EI_{y}$ $\left(10^{10}\;MPa\;mm^{4}\right)$	$EI_{x}$ $\left(10^{10}\;MPa\;mm^{4}\right)$	$G_{zy}A$ $\left(10^{7}\;MPa\;mm^{2}\right)$	$G_{zx}A$ $\left(10^{7}\;MPa\;mm^{2}\right)$
400	7.725	5.412	16.540	0.226	0.585
200	7.765	5.334	16.502	0.483	0.950
100	7.850	5.315	16.563	0.804	1.207
50	8.000	5.345	16.770	1.032	1.386
25	8.175	5.428	17.161	1.147	1.537
5	8.446	5.746	17.933	1.594	1.978
Analytically	7.686	5.231	16.323	1.236	1.375

**Table 2:** Stiffness of Z profile (without holes and no corner-rounding) with constant elongation depth of 100 mm, depending on mesh seed.

Seed $\left(mm\right)$	EA $\left(10^{7}\;MPa\;mm^{2}\right)$	$EI_{y}$ $\left(10^{10}\;MPa\;mm^{4}\right)$	$EI_{x}$ $\left(10^{10}\;MPa\;mm^{4}\right)$	$G_{zy}A$ $\left(10^{7}\;MPa\;mm^{2}\right)$	$G_{zx}A$ $\left(10^{7}\;MPa\;mm^{2}\right)$
15.0	7.87	5.32	16.59	0.818	1.217
12.5	7.86	5.32	16.58	0.815	1.215
10.0	7.86	5.31	16.57	0.812	1.212
7.5	7.85	5.32	16.57	0.806	1.209
5.0	7.85	5.32	16.56	0.804	1.207
2.5	7.85	5.31	16.55	0.802	1.206
Analytically	7.67	5.23	16.32	1.236	1.375

**Table 3:** Stiffness of C profile (without holes and no corner-rounding) with 5 mm mesh, depending on the elongation depth.

Depth $\left(mm\right)$	EA $\left(10^{7}\;MPa\;mm^{2}\right)$	$EI_{y}$ $\left(10^{10}\;MPa\;mm^{4}\right)$	$EI_{x}$ $\left(10^{10}\;MPa\;mm^{4}\right)$	$G_{zy}A$ $\left(10^{7}\;MPa\;mm^{2}\right)$	$G_{zx}A$ $\left(10^{7}\;MPa\;mm^{2}\right)$
400	9.54	3.77	15.6	0.215	0.596
200	9.58	3.63	15.5	0.566	1.029
100	9.67	3.57	15.5	0.989	1.376
50	9.84	3.58	15.7	1.283	1.639
25	10.1	3.62	16.0	1.438	1.859
5	10.4	3.80	16.8	1.988	2.423
Analytically	9.49	3.46	15.3	1.551	1.583

**Table 4:** Stiffness of C profile (without holes and no corner-rounding) with constant elongation depth of 100 mm, depending on mesh seed.

Seed $\left(mm\right)$	EA $\left(10^{7}\;MPa\;mm^{2}\right)$	$EI_{y}$ $\left(10^{10}\;MPa\;mm^{4}\right)$	$EI_{x}$ $\left(10^{10}\;MPa\;mm^{4}\right)$	$G_{zy}A$ $\left(10^{7}\;MPa\;mm^{2}\right)$	$G_{zx}A$ $\left(10^{7}\;MPa\;mm^{2}\right)$
15.0	9.69	3.576	15.54	1.01	1.39
12.5	9.68	3.576	15.53	1.00	1.39
10.0	9.68	3.578	15.53	0.996	1.38
7.5	9.67	3.575	15.52	0.994	1.38
5.0	9.67	3.570	15.51	0.989	1.38
2.5	9.66	3.555	15.49	0.987	1.37
Analytically	9.49	3.459	15.27	1.55	1.58

**Table 5:** Comparison of stiffnesses obtained from homogenization technique between full and with-hole profiles of Z, C profile and square tube.

	EA $\left(10^{7}\;MPa\;mm^{2}\right)$	$EI_{y}$ $\left(10^{10}\;MPa\;mm^{4}\right)$	$EI_{x}$ $\left(10^{10}\;MPa\;mm^{4}\right)$	$G_{zy}A$ $\left(10^{7}\;MPa\;mm^{2}\right)$	$G_{zx}A$ $\left(10^{7}\;MPa\;mm^{2}\right)$
Z profile	7.43	4.74	15.1	0.77	1.19
Z profile with hole	5.96	4.71	14.8	0.77	0.52
C profile	9.09	3.17	14.0	0.95	1.35
C profile with hole	7.68	2.71	13.9	0.95	0.75
Square tube	24.2	37.3	37.2	3.74	3.74
Square tube with hole	20.9	33.4	33.4	2.35	2.34

## Data Availability

The data presented in this study are available on request from the corresponding author.
